# Continuous Temperature Monitoring via Wearable Devices for Fever or Infection Management in Acute Care Hospitals: Scoping Review of Clinical Implementation and Data Analytics

**DOI:** 10.2196/89773

**Published:** 2026-09-11

**Authors:** Yuwei Liu, Qimeng Zhao, Ka Li, Dawn Dowding

**Affiliations:** 1 West China School of Nursing / West China Hospital Sichuan University Chengdu, Sichuan China; 2 Division of Nursing, Midwifery and Social Work, School of Health Sciences University of Manchester Manchester United Kingdom; 3 Nursing Key Laboratory of Sichuan Province Chengdu, Sichuan China; 4 Medicine and Engineering Interdisciplinary Research Laboratory of Nursing & Materials West China Hospital Sichuan University Chengdu, Sichuan China

**Keywords:** body temperature, fever, infections, mHealth, mobile health, scoping review, wearable device

## Abstract

**Background:**

Infections are a major health concern in hospitalized patients. Fever is an early sign of infection, making temperature monitoring essential for infection surveillance. Wearable devices are increasingly being explored for continuous temperature monitoring in acute care hospitals, but how temperature data from wearables are monitored, presented, and used to support fever or infection management in clinical practice remains poorly understood.

**Objective:**

This study aims to map existing evidence on the use of temperature data from wearables for fever or infection management in acute care hospitals, focusing on wearables’ characteristics; data transmission, storage, and presentation strategies; data preprocessing and analytic approaches; and the maturity of wearables’ clinical integration.

**Methods:**

We searched MEDLINE, Embase, Web of Science, CINAHL, and IEEE Xplore for publications from January 2013 to July 2026 using the population, concept, context (PCC) framework. Primary research studies using wearables to monitor patients’ body temperature in acute care hospitals for fever or infection management, published in English or Chinese, were eligible. Temperature data analyses were classified according to the descriptive, diagnostic, predictive, and prescriptive analytics framework. The clinical integration of wearables was assessed using a tailored maturity framework modified from the World Health Organization’s stages of maturity for digital health interventions.

**Results:**

We included 29 publications from 26 studies, published between 2018 and 2026. Eighteen wearable devices were identified, monitoring temperature at the axilla, chest, wrist, or upper arm. Continuous data streams were predominantly transmitted in real time, while 5 studies used non–real-time batch uploads, periodic synchronization, or device-memory downloads. Only 7 studies presented wearable temperature data in clinical settings, displaying data on mobile devices and/or centralized monitoring stations; only 1 study reported enabling alerts through electronic health records (EHRs). Invalid sensor data filtering methods varied across studies, such as physiological thresholds, firmware quality scores, and statistical outlier detection. Temperature data were most frequently analyzed by descriptive analytics (n=19) to depict the frequency, timing, and duration of fever episodes, followed by diagnostic analytics (n=6) to identify risk factors or distinguish causes of fever, and predictive analytics (n=9) to forecast impending fever or infections. None of the included studies used prescriptive analytics. For clinical integration, most studies were at the clinical validation stage (n=9) or clinical research stage (n=17), corresponding to an early stage of maturity; 3 studies reached the routine clinical practice stage, and none progressed to multicenter implementation or full integration stages.

**Conclusions:**

While promising, the clinical integration of wearables remains at an early stage of maturity. Critical gaps exist in translating temperature data into actionable clinical insights, with limited data presentation, poor EHR interoperability, and underdeveloped analytic approaches. Future research should prioritize standardized data cleaning frameworks, workflow integration, and clinical interpretation to facilitate the active use of wearable data in clinical decision-making.

**Trial Registration:**

OSF Registries osf.io/v6sp8; https://osf.io/v6sp8

**International Registered Report Identifier (IRRID):**

RR2-10.1136/bmjopen-2025-103630

## Introduction

Infections are a major health burden in hospitalized patients, particularly those acquired within health care settings, posing a significant threat to global patient safety [1]. An estimated 5.4% to 7.8% of patients in acute care hospitals across Europe [2] and 12% to 18% in the Eastern Mediterranean Region [3] are affected by health care–associated infections. Timely recognition and earlier treatment of infections are crucial for reducing morbidity and mortality [4]. As one of the earliest and most apparent symptoms, fever is a vital diagnostic and monitoring indicator that reflects the clinical course of many infectious diseases [5]. Therefore, temperature monitoring serves as a fundamental component of infection surveillance and management, supporting clinical decisions on whether to initiate a fever workup or prescribe antibiotics.

Traditional temperature measurement mainly relies on intermittent manual recording by nurses. However, the intervals between observations cannot fully capture temperature changes and may miss febrile events or delay the detection of infections [6]. In recent years, wearable technologies have enabled continuous temperature monitoring in acute care hospitals [7]. Wearable devices are electronic sensors designed to be directly worn on the body to perceive and record various physiological parameters [8], encompassing a range of sensor-based devices such as patches, rings, wristbands, and smartwatches. Featuring wireless connectivity, real-time data collection, and remote monitoring capabilities, wearables have attracted growing interest for clinical applications. Recent studies have explored the accuracy and feasibility of wearables for temperature monitoring in diverse clinical settings, such as surgical wards, emergency departments, oncology wards, and intensive care units (ICUs) [9].

The high-frequency temperature data generated by wearables contain rich physiological information, which holds the potential to transform infection care by providing data-driven insights into inflammatory responses and infection trajectories [10]. With advanced analytical approaches, these data can be used to derive temperature features that capture temporal dynamics such as trends, patterns, and circadian rhythms. These features could serve as digital biomarkers to support early detection of febrile events, personalized alerts, and timely interventions [11]. For instance, a fever detection algorithm using a chest patch captured fever episodes 4.3 hours earlier than clinical assessments in patients at high risk of febrile neutropenia [12]. Vargas et al [13] demonstrated that entropy analysis of continuous body temperature could distinguish bacterial infections from other causes of fever, potentially enabling earlier antibiotic cessation. More broadly, wearable data are increasingly integrated into AI predictive models of infectious disease within real-time surveillance and clinical decision support ecosystems [14].

However, translating this potential into practice remains hindered by considerable hurdles when deploying wearables within the complex, high-noise environments of acute care hospitals. Wearable data collected in such environments typically contain various anomalies, such as missing values, outliers, motion artifacts, random noise, recording errors, and redundant data [15]. Without effective filtering, these anomalies may trigger false alerts and contribute to alert fatigue. Although wearables technically streamline routine temperature measurement and reduce time and effort [16], they may counterproductively increase nurses’ workload without proper clinical integration [17]. Moreover, while continuous monitoring captures subtle physiological fluctuations, difficulties in interpreting the sheer volume of temperature data often prevent these data from being translated into actionable clinical insights [18], resulting in a paradoxical situation where cognitive overload and data waste coexist. Given these limitations, wearables may simply be used as tools for passive data collection rather than active clinical decision support [19], with their potential remaining largely underexplored.

To better integrate wearables into clinical practice, there is a need to incorporate wearable data into existing clinical workflows and facilitate data interpretation to support clinical decision-making. However, most current studies remain focused on evaluating the accuracy and feasibility of wearables in clinical care [20]. While existing literature reviews have summarized available wearables and their clinical validation findings [9,21], there is a lack of synthesized evidence on how temperature data from wearables are processed, presented, and interpreted in clinical practice. For the next step, it is essential to shift the research focus from device performance assessment toward proactive data usage in clinical contexts. Therefore, to bridge the gap between technical feasibility and clinical utility, this study aims to review evidence on the use of wearables for continuous temperature monitoring in acute care hospitals to support fever or infection management, focusing on strategies for data transmission, presentation, and use, and the overall maturity of these wearables’ clinical integration.

## Methods

### Overview

We chose a scoping review because it provides the flexibility to synthesize heterogeneous evidence across different wearable device types, clinical contexts, and data analytical methods in this rapidly evolving field. We conducted the review following the Joanna Briggs Institute (JBI) manual for scoping reviews [22] and reported it in alignment with the PRISMA-ScR (Preferred Reporting Items for Systematic Reviews and Meta-Analyses extension for Scoping Reviews) guidelines [23]. The protocol was registered on the Open Science Framework (OSF) [24] and published [25] ahead of this review. The PRISMA-ScR checklist is provided in Multimedia Appendix 1.

### Research Question

Our review addressed the following questions:

- Question 1: what types of wearable devices have been used in acute care hospitals to monitor body temperature for fever or infection management?
- Question 2: how are the temperature data from wearable devices monitored, transmitted, and presented in acute care hospitals?
- Question 3: what analytical approaches have been used to process temperature data from wearable devices to support the management of fever or infections?
- Question 4: to what extent have these wearable devices been integrated into clinical practice?

### Selection of Studies

We adopted the Population, Concept, Context (PCC) framework to guide the development of search strategies and eligibility criteria.

- Population: hospitalized patients who are confirmed, suspected, or at risk of developing fever or infections.
- Concept: wearable devices that are capable of wireless, continuous, noninvasive body temperature monitoring by being worn on the external body surface [8]; fever was defined as a pathological elevation in the body’s set-point temperature [26,27]; infections refer to the invasion and multiplication of microorganisms, including bacteria, viruses, fungi, or parasites, in body tissues. We define continuous monitoring as automated time-series data acquisition at a predefined sampling frequency in real time or near real time (eg, every 1 minute or 5 minutes) [28], without manual initiation of each measurement.
- Context: acute care hospitals, which provide short-term, hospital-based care for patients with acute illness or injury. All inpatient wards and emergency departments were eligible.

The MEDLINE, Embase, Web of Science, CINAHL, and IEEE Xplore databases were searched from January 2013 to July 2026, as bibliometric analysis indicates a rapid increase in research on wearable technologies for health monitoring since 2013 [29,30]. The search was initially conducted in February 2025 and updated in July 2026 (both by reviewer YL). Search terms were organized into categories, including “wearable*,” “body temperature,” “fever,” “infecti*,” “patient*,” and “hospital.” Specific brands of wearables were identified from previous reviews and added to search strings [7,9]. Gray literature, including conference proceedings, preprints, and dissertations, was also searched. The detailed search strategies for databases and websites for gray literature are provided in Multimedia Appendix 2. The full search strategy was reviewed by a professional medical librarian using the PRESS (Peer Review of Electronic Search Strategies) checklist [31].

We uploaded the search results to Rayyan [32] to remove duplicates and conduct online screening. During the pilot screening, we compiled a preliminary list of potential temperature monitoring devices, discussed which qualified as wearables, and reached consensus on the eligible boundaries before formal screening. Then, a 2-step process, including title and abstract screening and full-text screening, was used. One reviewer (YL) screened all literature, with 20% randomly selected and independently screened by a second reviewer (QZ) to assess screening consistency. The interrater agreement using Cohen κ coefficients was 0.778 and 0.746 for each screening step, indicating substantial agreement. Disagreements were resolved through discussion with a third reviewer (DD). For multiple records derived from the same study, such as conference abstracts and research protocols, the formally published article was retained. The reference lists and citation lists of eligible articles and relevant literature reviews were manually searched to identify additional publications.

Studies were included if they used wearables to monitor patients’ body temperature in acute care hospitals for the purpose of fever or infection management, including but not limited to detection, diagnosis, prediction, treatment, or prognostic assessment. Primary research studies published in English or Chinese were eligible regardless of study design or publication type. Studies were excluded if they focused on monitoring local tissue temperature (eg, wound or foot), reported the technical design or development of wearables, assessed device accuracy exclusively without clinical interpretation of temperature data, or focused on the prevalence of epidemic infectious diseases rather than clinical care.

### Data Extraction and Analysis

Data were extracted from the main text and supplementary materials using a predefined data extraction form (Multimedia Appendix 3), including bibliographic and contextual attributes, wearable device characteristics, and strategies for data transmission, storage, and presentation. We also extracted temperature data analysis strategies, including data preprocessing, derived temperature features, and analytic methods. We classified temperature data analyses according to the descriptive, diagnostic, predictive, and prescriptive analytics framework [33,34], and further assessed the maturity stage of wearables’ integration in clinical practice with reference to the World Health Organization’s (WHO) manual, “*Monitoring and Evaluating Digital Health Interventions*” [35]. We also identified the role of nurses by reviewing the main text, author lists, and affiliations. We did not assess the methodological quality of the included publications in an attempt to map all available evidence, and quality appraisal is not mandatory in a scoping review according to JBI methodology [36]. Data extraction was performed by one reviewer (YL). A second reviewer (QZ) independently extracted data from a random 20% of the included publications to evaluate the reliability of the data extraction process. Discrepancies were discussed to reach a resolution, and a third researcher (DD) was consulted for unresolved discrepancies. A descriptive synthesis of the findings was presented in tables and figures.

## Results

### Study Selection

**Figure 1:**
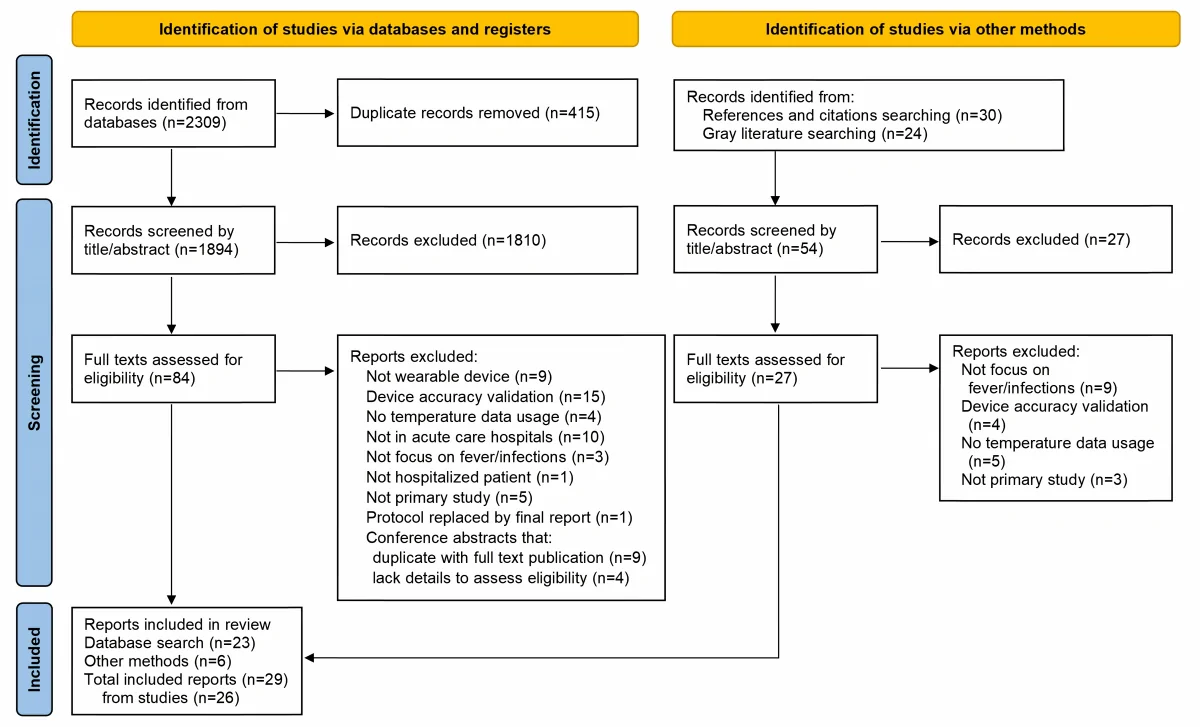
PRISMA (Preferred Reporting Items for Systematic Reviews and Meta-Analyses) flow diagram.

A total of 2309 records were identified through database searches. After duplicate removal (n=415), 1948 records (1894 from database searches and 54 from manual identification) were screened by title and abstract. Subsequently, 111 records (84 from database search screening and 27 from other methods) underwent full-text screening. We included 29 publications (from 26 studies) in this review. Figure 1 presents the study selection process. Three publications [6,37,38] reported data derived from the same patient cohort in one study, while 2 publications [39,40] analyzed data from another study.

### Study Characteristics

The 29 included publications (from 26 studies) comprised 25 journal articles (including 1 research letter with original data) and 4 conference abstracts, published in English (28/29, 97%) and Chinese (1/29, 3%) between 2018 and 2026. The included studies were conducted in Asia (9/26, 35%), Europe (8/26, 31%), North America (6/26, 23%), and Oceania (3/26, 12%). The majority were prospective observational studies (20/26, 77%), while 3 were retrospective observational studies, 1 was a randomized controlled trial, and 2 observational studies did not specify whether they were prospective or retrospective. Most studies were single-center (24/26, 92%), with only 2 studies being multicenter. Study populations were adult patients (17/26, 65%), pediatric patients (5/26, 19%), women in labor or pregnancy (3/26, 12%), and older adult patients (1/26, 4%). Clinical settings comprised oncology wards (9/26, 35%), surgical wards (6/26, 23%), medical wards (6/26, 23%), prenatal or delivery rooms (3/26, 12%), and ICUs (2/26, 8%). The primary events of interest were febrile events (15/26, 58%; including fever, febrile neutropenia, cytokine release syndrome [CRS], and intrapartum fever) and infections (11/26, 42%; including sepsis, pneumonia, intraamniotic infection, postoperative infectious complications, and COVID-19).

### Wearable Device Characteristics and Monitoring Parameters

**Figure 2:**
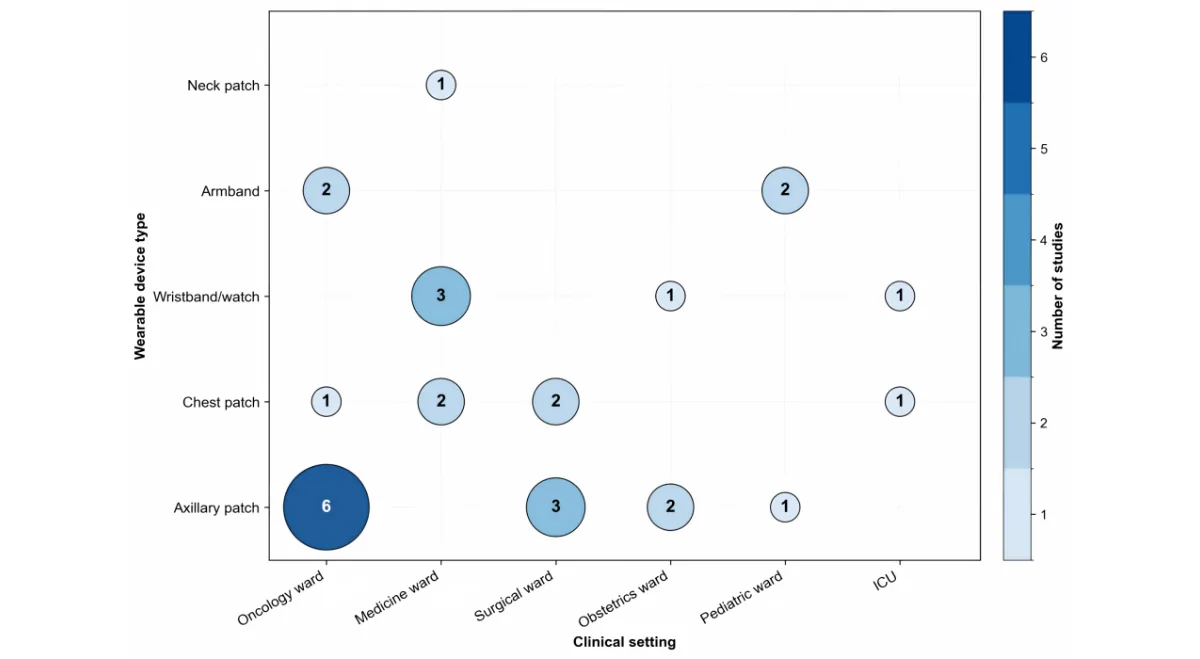
Number of included studies by clinical setting and wearable device type. Bubble size and color intensity represent study count for each combination. Studies using 2 wearable devices were counted under both types. ICU: intensive care unit.

A total of 18 wearable devices were identified (Table 1), including 4 chest patches, 6 axillary patches, 2 smartwatches, 2 wristbands, 3 upper arm or leg bands, and 1 neck patch. Most devices (11/18, 61%) monitored multiple physiological parameters, while 7 devices were dedicated temperature sensors. Seven devices measured axillary temperature, while 11 devices measured skin temperature at various sites, including the chest (n=3), wrist (n=4), upper arm or leg (n=3), and neck (n=1). Five devices provided estimated core temperature (VitalPatch, CORE, Everion, Moni-Patch, and iThermonitor), with measurements derived from axillary or skin temperature readings. Axillary patches were the most frequently used device, with the highest number of studies reported in oncology wards (Figure 2).

Among the 18 identified wearables, 78% (14/18) were medical-grade and 11% (2/18) were consumer-grade, while the grade was not specified for 2 devices. Ten wearables were approved by the US Food and Drug Administration (FDA), with the majority (8/10) approved as class II devices for use by health care professionals on general patients in health care settings, while 2 were approved for home use or ovulation tracking (Multimedia Appendix 3). However, reporting on the regulatory status and clinical validation of wearables showed gaps. Among the included publications, only 41% (12/29) reported the device grade, and just 35% (10/29) confirmed FDA approval or Conformité Européenne (CE) marking. Only 35% (10/29) of publications validated the wearables by comparing the temperature data with standard clinical measurements as part of their results, and 17% (5/29) cited previously published validation findings. One article noted that the wearable device used in their study had not yet been clinically validated [41], and 2 articles mentioned manufacturer preclinical validation [42,43]. The remaining publications (11/29, 38%) did not mention clinical validation.

**Table 1:** Characteristics of wearables identified from the included studies.

Wearable sensor; grade; regulatory approval	Physical location	Physical metrics	Monitoring frequency	Battery life
TempTraq TT-100; medical; FDA^a^/CE^b^	Axilla	Axillary temperature	10 seconds^c^; 2 minutes^d^	1 day^e^
iThermonitor; medical; FDA	Axilla	Axillary temperature (estimate core temperature)	4 seconds^d^	30 days
Lifetemp; medical^e^; CE^e^	Axilla	Axillary temperature, heart rate, and respiratory rate intervals	1 minute^d^	5 days^e^
SteadyTemp; medical^e^; FDA/CE^e^	Axilla	Axillary temperature	5 minutes^d^	10 days^e^
thynC MT100D; medical^e^; MFDS^e,f^	Axilla	Axillary temperature	1 minute^d^	Not reported
FeverScout; medical^e^; FDA/CE^e^	Axilla	Axillary temperature	15 seconds^c^; 2 minutes^d^	Rechargeable^e^
SensiumVitals; medical; CE	Chest patch with an axillary sensor	Axillary temperature, heart rate, and respiratory rate	2 minutes^d^	5 days
VitalPatch; medical; FDA/CE^e^	Chest	Skin temperature, Core temperature, heart rate, heart rate variability, and respiratory rate	1 minute^d^	5 days^e^
Biobeat BB-613WP; medical; FDA	Chest	Skin temperature, heart rate, blood pressure, respiratory rate, cardiac output, cardiac index, and SVR^g^	15 minutes^d^	6 days
Biosensor Voyage; not reported; not reported	Chest	Skin temperature, heart rate, and respiratory rate	30 seconds^d^	3 days
HEARThermo wristband; not reported; not reported	Wrist	Skin temperature and heart rate	10 seconds^d^	Not reported
Ava bracelet; medical^e^; FDA^e^	Wrist	Skin temperature, pulse rate, heart rate variability, and respiratory rate	10 seconds^d^	Rechargeable
HUAWEI WATCH 3; consumer; N/A^h^	Wrist	Skin temperature, heart rate, respiratory rate, SpO_2_^i^, and cough sound	25 Hz^c^	Not reported
Empatica E4 Watch; medical; FDA^e^	Wrist	Skin temperature, pulse rate, respiratory rate, electrodermal activity, SpO_2_, and activity	4 Hz^c^	3 days (rechargeable)
Everion; medical; FDA/CE	Upper arm	Skin temperature, core temperature, heart rate, heart rate variability, respiratory rate, electrodermal activity, SpO₂, blood pressure wave, and activity	1 second^c^; 1 minute^d^	Rechargeable
CORE; consumer; N/A	Upper arm or chest	Core temperature	1 second^d^	Rechargeable
Gen2; medical; FDA	Upper arm	Skin temperature, respiratory rate, pulse rate, SpO₂, motion, and blood pressure	2 seconds^d^	Rechargeable^e^
Moni-Patch; medical^e^; PMDA^e,j^	Neck	Core temperature	Not reported	Not reported

^a^FDA: US Food and Drug Administration.

^b^CE: Conformité Européenne.

^c^Indicates sampling frequency.

^d^Indicates recording frequency.

^e^Information not reported in the included studies was obtained from official regulatory databases/websites and manufacturers’ websites.

^f^MFDS: Ministry of Food and Drug Safety, Republic of Korea.

^g^SVR: systemic vascular resistance.

^h^N/A: not applicable.

^i^SpO_2_: peripheral oxygen saturation.

^j^PMDA: Pharmaceuticals and Medical Devices Agency, Japan.

There was variation in monitoring frequency and duration across studies. Body temperature was monitored at varying intervals, including every 1, 2, 4, 10, or 30 seconds; every 1, 2, 5, or 15 minutes; or at sampling frequencies of 4 Hz or 25 Hz. The median duration of temperature monitoring per patient ranged from 3 hours to 20 days across studies. Battery life across the included wearables ranged from 1 to 30 days. Holt et al [44] reported that, despite a theoretical maximum battery life of 3 days, the device recorded data only up to 44 hours in practice. To meet the requirements of clinical monitoring durations, different strategies were used depending on the device, such as daily charging [41,45,46], battery replacement [47], or regular device change every 24 hours [39,42,48], 3 days [49,50], or 6 days [51].

### Temperature Data Transmission, Storage, and Presentation

#### Data Transmission

**Figure 3:**
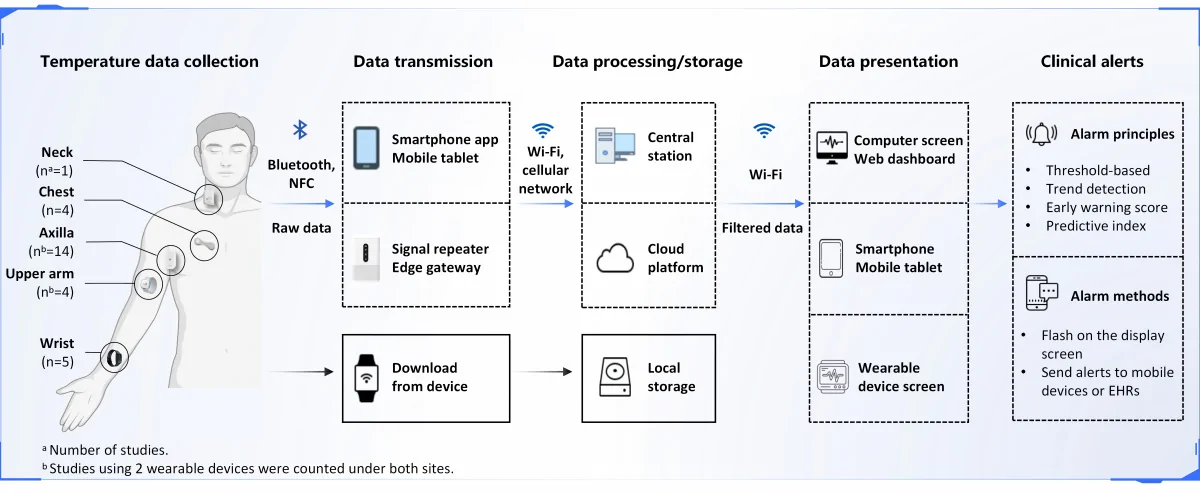
Data transmission, storage, and presentation in clinical practice.

Figure 3 illustrates the data transmission strategies. Wearable data were predominantly transmitted through a wireless pipeline (20/26, 77%), initially from sensors via Bluetooth to nearby mobile devices (smartphones, tablets, or iPads), engineered bridges, signal repeaters, or transportable gateways. Subsequently, 16 studies described the further transmission of data from mobile devices or bridges to cloud service platforms (n=11) or central computers in the wards (n=5). These data were typically transmitted in real time or as continuous streams. Data latency was quantitatively reported in only 1 feasibility study [46], where data arrived at the dashboard with a median delay of 5 minutes for Everion and 2 minutes for CORE. After addressing transmission bottlenecks caused by 2 wearables competing on a single gateway, the median delay for both devices decreased to 0 minutes (range 0-2 minutes). Kim et al [52] used a dual-gateway wireless architecture that allowed the patch to communicate simultaneously with 2 nearby gateways, thereby ensuring uninterrupted transmission with minimal data latency or signal loss.

In contrast, 19% (5/26) of studies used non–real-time data transmission methods, which included a batch upload strategy with 10-minute intervals [48], synchronizing temperature data each morning via a smartphone app following overnight monitoring [43], reading out patch data via near-field communication (NFC) every 24 to 72 hours using a smartphone app [53], or downloading data from the devices’ internal memory after regular removal [44,45]. The remaining 1 study did not specify data transmission strategies or frequency.

#### Data Storage and Protection

Four studies reported that data could be stored temporarily on the device for 18 hours [54], 90 hours [45], or up to 6 days [55] with time stamps [47]. This feature supported local data retention during periods without network coverage, with buffered data automatically synchronizing upon connection restoration. Eight of the studies [41,46,50,54,56-59] reported that wearable data were stored on cloud platforms, with 2 studies [41,46] irreversibly deleting data from the cloud dashboard after they were transferred to a local research database. Four studies [47,52,55,60] stored wearable data on local central workstations or hospital computer systems. One study [45] reported that data were stored on the device, downloaded via a stationary Bluetooth interface for local storage, without using a web application due to regulatory requirements. Reporting on data security remains poor: only 38% (10/26) of studies described privacy protection strategies, including access restrictions [17,50,55,56], data anonymization [39], encryption for data in transit and data stored [41,46], local data storage [45], and the use of a secure wireless network and secure communication protocols [52]. Among these, 2 studies [45,49] reported alignment with the European Union (EU) General Data Protection Regulations.

#### Data Presentation in Clinical Practice

Only 27% (7/26) of studies presented temperature data from wearables in clinical settings, displaying data on mobile devices (n=5) and/or centralized monitoring interfaces (n=6). Among these, 4 studies [17,51,54,61] used multiple interfaces, displaying data both on mobile devices carried by nurses and on ward computer screens [17,61] or web-browser–based dashboards enabling remote access [51,54]. In contrast, 3 studies used a single interface, visualizing temperature curves either on a central computer screen in the nurse station [47,60] or on a paired bedside tablet [62]. Two studies reported that the wearable systems could send digital alerts to health care professionals via smartphones [17,61] or electronic health records (EHRs) [17]. Apart from the single study allowing alerts through EHRs [17], no other studies reported integration with EHR systems.

Conversely, 42% (11/26) of studies did not present wearable data in clinical settings, using the devices solely for research data collection. Among these, 9 studies explicitly stated that wearable data were blinded to clinical staff [41,45,48,50-53,58,59] to avoid influencing clinical decisions [46], while 2 studies [44,45] directly downloaded data from the device without dashboards. In 1 study [49], although heart rate, respiratory rate, and peripheral oxygen saturation (SpO_2_) were available in real time for caregivers to support care, skin temperature was not directly available because its clinical relevance was considered uncertain. The remaining studies (8/26, 30.8%) lacked sufficient information to determine whether data were displayed or provided to clinical staff.

### The Use of Temperature Data

#### Data Quality Assessment and Preprocessing

The reporting of missing sensor data varied across the studies. Only 62% (16/26) of studies reported the reasons and proportions of missing temperature data, while 2 studies indicated that no missing data were observed [47,60]. In the remaining 31% (8/26) of studies, missing data were not mentioned. The reported causes for missing data were grouped into 4 main categories: patient factors (eg, discomfort, nonadherence to the wearing protocol, and improper handling), device factors (eg, device malfunction and battery issues), wearing quality (eg, device detachment, poor skin contact, and sweating), and signal factors (eg, synchronization failure, motion artifacts, and patients moving out of signal coverage). The metrics used to quantify missing data also varied. Five studies described the hours with low-quality or missing data (ranging from 3.6% to 11.7%), 5 studies reported the hours with valid data (ranging from 67% to 99%), while 6 studies reported the cases with missing values (ranging from 2% to 31.4%).

Several methodologies to detect invalid data were identified from the included studies, including heuristic filtering based on physiological plausibility thresholds, firmware-integrated quality indexing, and statistical outlier detection frameworks. Six studies used thresholds to filter out implausible values (eg, below 35.5 °C or 35 °C for axillary temperature [39,40,50,52] or 31 °C for skin temperature [44]), with one of them involving patients with terminal illness using a lower threshold of 25 °C for skin temperature [49]. Four studies [41,45,46,51] reported that wearables’ firmware generated a quality score for temperature data per minute, though the use of the quality score differed. In the study by Garbern et al [54], a quality score was calculated based on signal quality each minute; windows with quality scores below 75% were labeled as low quality and excluded from analysis. In contrast, Jacobsen et al [45] did not use the quality score to select data; instead, they retained only periods with at least 3000 data points per hour for analysis. Eisenkraft et al [51] picked outliers with a difference from the mean exceeding the Q3 + 3 × IQR threshold. Additionally, the initial 5 minutes of data were often excluded due to device warm-up and stabilization periods [49].

Strategies for handling invalid or missing sensor data were heterogeneous across studies, spanning data filtering, smoothing, imputation, and native algorithmic handling. Some studies excluded entire cases [43,44,50,52,53,56] when extensive missingness occurred (eg, patch fell off over 6 hours), or targeted removal of data points identified as anomalies [17,44,50,51,53,54]. For smoothing, van Goor et al [49] computed 15-minute median values to reduce the impact of short-lasting outliers and missing data. For imputation, Rajeeve et al [59] applied forward-fill methods to address missing sensor data, whereas Debnath et al [40] retained the dropped data without imputation, considering the imputation process may introduce potential bias. Finally, 2 studies embedded missing-data handling directly within their modeling pipeline. Chen et al [56] used extreme gradient boosting (XGBoost), which handles missing values during training by automatically learning the optimal split direction; Jacobsen et al [45] used end-to-end deep learning algorithms that could handle artifacts and data gaps without extra preprocessing.

#### Temperature Features Derived From Wearable Data

Three major categories of temperature features were derived from raw continuous readings, including basic descriptive statistics, threshold-based features, and temporal features. Basic descriptive statistics were the most commonly used features, including the mean (n=14), median (n=3), maximum body temperature (n=5), SD (n=5), variance, and coefficient of variation (n=1). These features were used to characterize the temperature dataset and to compare temperature measurements across different monitoring methods, patient cohorts, and anatomical sites. For example, Liu et al [47] compared the peak temperature monitored by continuous monitoring and intermittent monitoring, finding that continuous monitoring captured 0.29 °C higher peak values.

The second major category was threshold-based temperature features, which were identified in 21 publications. These studies used various predefined cutoffs (eg, 37.2 °C, 37.8 °C, 38 °C, and 38.5 °C) to identify fever, subsequently using continuous temperature data to quantify the features of fever episodes, including frequencies, the cumulative duration [62] or percentage of time spent above the threshold [54], and severity (quantified by calculating the area under the time-temperature curve [62]). Notably, Rajeeve et al [59] reported that individualized axillary temperature thresholds (baseline mean + 2 SD) combined with a fixed threshold (36.4 °C) enabled earlier detection of temperature elevation before clinical recognition of CRS episodes than a simpler fixed 38.0 °C threshold did. Four studies [17,51,54,61] relied on thresholds set by early warning score (EWS) systems to trigger alerts when body temperature deviated from the preset physiological range. Eight studies focused on the timing of fever detection [6,39,42,47,50,52,58,60]. For instance, Sampson et al [42] compared the differences in the timing of fever detection, showing that continuous temperature monitoring detected fever events 12 hours earlier than standard monitoring. Wang et al [60] used 24-hour continuous recordings and a brute-force strategy to simulate different intermittent monitoring schedules, identifying the combination with the highest fever detection rate.

Ten publications further incorporated temporal features of body temperature, including trends, trajectory patterns, and circadian rhythmicity. Five studies explored visual trends and patterns: 3 studies observed rising temperature trends associated with infections [6,51,62]; 2 studies tried visual inspection of temperature curves in the 48 hours preceding fever episodes, although they did not yield clinically significant predictive information [41,46]. A more computational pattern recognition technique was used by Ren et al [37], who used dynamic time-warping to characterize temporal trajectory patterns to differentiate between fever etiologies. Another 3 studies focused on circadian rhythmicity, either by modeling the rhythm with cosinor models to detect a loss of circadian variation in skin temperature among patients who were critically ill with COVID-19 [49], or by using residual deviations from the patient's individual circadian rhythm pattern to predict febrile adverse events [6,45]. Two studies developed machine learning models using temperature data as inputs, with time-series features generated by aggregating temperature data into rolling windows of 6 to 14 hours in one study [59], while the other used a fixed 12-hour window [63].

#### Classification of Temperature Data Analytics

An established data analytics framework categorizes 4 analytic approaches by the questions they address: “What happened?” (descriptive), “Why did this happen?” (diagnostic), “What might happen in the future?” (predictive), and “What should be done next?” (prescriptive) [34]. Using this framework, we classified the temperature data analyses in the included publications as descriptive (used in 19/29, 65% of studies), diagnostic (used in 6/29, 21% of studies), and predictive (used in 9/29, 31% of studies); none used prescriptive analytics (Table 2). Five publications used multiple analytic approaches.

**Table 2:** Classification of data analytics for wearable-generated temperature data.

Types of analytics and description of the included studies			Temperature features		Analysis techniques
**Descriptive analytics (n=19)**					
	Compared differences in temperatures recorded across monitoring methods, patient cohorts, or anatomical sites [43,44,47]	Mean, SD, median, and maximum		Student *t* test and ANOVA	
	Counted the numbers and frequencies of febrile events and compared the time difference between fever episodes detected by continuous and intermittent monitoring [17,38,39,42,47,48,50,52,58,61]	Timing of fever		Student *t* test	
	Developed a heuristic scoring system to quantify the clinical relevance of fever detection, incorporating fever duration (short spike <60 minutes; prolonged episode ≥60 minutes) and clinical actions triggered [53]	Thresholds and fever duration		Descriptive comparison	
	Hourly points from the continuous temperature data were used to simulate 1 to 24 intermittent daily measurements to identify the optimal timing and frequency achieving the highest fever detection rate [60]	Counts and frequencies		Brute-force strategy	
	Compared the performance of fixed, individualized (baseline mean + 2SD), and combined thresholds for axillary temperature in detecting cytokine release syndrome [59]	Thresholds		Grid search	
	Detected abnormal values or temperature deviations from preset thresholds to trigger alerts [17,61]	Thresholds		Described the counts and frequencies	
	Visualized patterns of temperature, such as rising trends or fluctuations in temperature curves [41,46,51]	Visual patterns		Line plots	
	Described the disappearance of the circadian rhythm of skin temperature in patients with critical illness, in comparison with recovered patients [49]	Circadian rhythmicity		Cosinor models	
**Diagnostic analytics (n=6)**					
	Classified fevers of infectious or non-infectious causes using temperature trajectory patterns within 4 hours around fever start time [37]	Trajectory patterns		Dynamic time warping and k-means clustering	
	Quantified fever intensity and evaluated its association with epidural analgesia duration and dosage to identify risk factors for fever [62]	Area under time-temperature curve		Logistic regression and ROC^a^ curve analysis	
	Examined the correlation between skin temperature and oral/ear canal temperature and identified a diagnostic cut-off value of skin temperature for detecting fever or infections [44,48,57]	Correlations		ROC curve analysis	
	Analyzed the correlation between standard deviation and peak temperature to reveal an increased temperature variability before and after the spike [39]	Correlations		Pearson correlation and linear regression	
**Predictive analytics (n=9)**					
	Predicted SCC^b^ by inputting temperature data into a deep neural network to calculate an SCC score reflecting dissimilarity from normal patterns [45]	Cosine similarity		Deep neural network	
	Used aggregated temperature features (median, variance, coefficient of variation) as inputs for pulmonary infection prediction models [56]	Median, variance, coefficient of variation		XGBoost^c^	
	Extracted residual deviations of temperature from the patient's individual circadian rhythm pattern as early signs of febrile adverse events [6]	Circadian residuals		Circadian modeling and genetic algorithm	
	Used continuous temperature data as inputs for machine learning models to predict the risk of advanced sepsis [54], cytokine release syndrome [59], or COVID-19 deterioration within the next 24 hours [63]	Mean, SD, extreme value, and percentage of time spent over thresholds		Machine learning models	
	Used skin temperature features (eg, mean and maximum) within various time windows before fever onset as early predictive signals for impending fever episodes [40,55,57]	Correlations		Logistic regression and generalized estimating equation	

^a^ROC: receiver operating characteristic.

^b^SCC: serious clinical complications.

^c^XGBoost: extreme gradient boosting.

Descriptive analytics were used in 18 publications. Descriptive analytics describe the temperature data using basic descriptive statistics such as means, frequency, and variation, and present the data through histograms, box plots, scatter plots, and line charts. These studies reported that continuous temperature monitoring via wearables enabled earlier fever detection [17,38,39,42,47,48,50,58,61], captured higher temperatures [43,44,47], detected more febrile events [38,42], and yielded higher fever detection rates [52,60] than conventional intermittent monitoring, suggesting the potential of wearables to improve fever or infection detection.

Diagnostic analytics were used in 6 publications. Diagnostic analytics involve comparing coexisting trends, uncovering correlations between variables, and determining causal relationships where possible to identify the risk factors or causes of fever. Pearson correlation, linear regression, and logistic regression were used to explore associations between temperature variables [39,57] and identify risk factors for fever [62]; receiver operating characteristic (ROC) analysis was used to derive diagnostic cutoff values for fever based on wearable skin temperature [44,48]; and k-means clustering was used to classify fevers of infectious or noninfectious causes [37]. These studies highlighted the potential of wearable data to support clinical diagnosis related to fever or infections.

Predictive analytics were identified in 9 publications. By using historical data and statistical modeling, predictive analytics identify patterns and predictors of future events and forecast the probability of fever or infections. Methods included logistic regression, machine learning models, and circadian modeling, with temperature features serving as predictive inputs. Six studies developed predictive models to estimate the risk of pulmonary infection [56], febrile adverse events [6,45], CRS [59], deterioration [63], and advanced sepsis [54]. Another 3 studies [40,55,57] focused on identifying skin temperature features within various time windows before fever onset as early predictive signals for impending fever episodes. These findings suggested the potential of wearables to enhance personalized fever or infection risk prediction.

Prescriptive analytics uses algorithms with “if” and “else” rules to analyze data and recommend optimal next steps or actions. In health care, this approach can be operationalized through clinical decision support systems that provide therapeutic recommendations, for example, whether medication or physical examinations are required. However, none of the included studies used prescriptive analytics.

### Maturity Stages of Wearable Device Integration in Clinical Practice

Table 3 maps the original WHO stage of maturity for digital health interventions and our modified maturity framework tailored to wearables, outlining 6 phases in a continuum. As specified in the eligibility criteria, studies focused on prototype design, preclinical testing, or only on validating the accuracy of wearables were excluded. Among the 29 included publications, most were classified as being at the clinical validation stage (stage 3a: 9/29, 31%) or clinical research stage (stage 3b: 17/29, 59%). The primary objectives of these studies were to evaluate the performance of wearables for monitoring body temperature in clinical settings or to collect research data, corresponding to the pilot stage in the WHO framework. In 3 studies, wearables were applied in routine clinical practice (stage 4: 3/29, 10%) as a supplement to standard temperature monitoring by nurses, aligning with the demonstration stage. None of the studies in this review had progressed to multicenter implementation or full integration.

**Table 3:** Maturity stages of wearable devices’ integration in clinical practice, modified from WHO^a^ “Monitoring and Evaluating Digital Health Interventions.”

WHO stage of maturity and modified stage of maturity				Publications		Focus on wearable devices at each stage
**Early**						
	**Preprototype**					
		Stage 1: proof of concept	Not included		Hypothesis building, clinical needs/context assessment, preliminary solution modeling, and initial technical feasibility testing	
	**Prototype**					
		Stage 2: preclinical test	Not included		Technical design, functionality assessment, and testing of technical stability (eg, device compatibility, data transmission range, and system robustness) in simulated or controlled environments	
	**Pilot**					
		Stage 3a: clinical validation	n=9		Validation of accuracy, precision, and feasibility in real clinical settings, typically via field tests and clinical trials using gold standards for comparison [39,41,42,46,47,50,52,58,61]	
		Stage 3b: clinical research	n=17		Wearables are used exclusively for data collection in research, with the data collected not being provided to support clinical practice [6,17,37,38,40,43-45,48,53-57,59,62,63]	
**Mid**						
	**Demonstration**					
		Stage 4: routine clinical application	n=3		Wearables are integrated into routine clinical workflows as a supplement to standard care; wearable system operating independently from the hospital’s EHR^b^ [49,51,60]	
**Advanced**						
	**Scale-up**					
		Stage 5: multicenter implementation	n=0		Wearables are adopted as part of routine clinical practice across multiple centers or scaled up to subnational, national, or population levels.	
	**Integrated and sustained program**					
		Stage 6: full integration	n=0		Full integration into clinical practice, with EHR interoperability, use in decision support systems, and supportive policies and financing enabling broader health system incorporation.	

^a^WHO: World Health Organization.

^b^EHR: electronic health record.

### Nurses’ Roles

The findings show that nurses were engaged in both research and clinical practice related to wearable devices. As researchers (identified in 7 studies), nurses acted as principal investigators who led and conducted the research [47,48,50,55,57,61] or served as clinical research nurses responsible for patient recruitment, device maintenance, and data collection [54,61]. In clinical practice, 12 studies described nurses as direct users of wearable devices for temperature monitoring, evaluating temperature data generated by the devices, and responding to device alerts.

## Discussion

### Principal Findings

This scoping review assessed 29 publications from 26 studies using wearables to monitor body temperature in acute care hospitals for fever or infection management. We identified a variety of wearables used across diverse patient populations and clinical settings since 2018, indicating growing interest in this emerging technology despite its recent introduction to clinical practice. By mapping the current evidence on wearable temperature data pipelines from sampling through transmission and presentation to clinical use, we also identified overall limited clinical integration and critical gaps at each stage. These findings may serve as a practical reference for the design and implementation of wearable-based monitoring systems within clinical workflows and highlight barriers to be addressed to move toward more sustainable clinical adoption.

### Different Types of Wearables and Clinical Utility of Their Temperature Data

This review identified a preference for medical-grade devices in clinical settings, while consumer-grade devices predominated in home-based remote monitoring in oncology [20] or cardiovascular care [64]. This preference reflects the need for accurate temperature data in clinical practice, which influences the selection of wearables and how the data can be used. Wearables generally provide temperature readings by monitoring thermal activity at the skin surface, which is susceptible to ambient conditions and skin perfusion [65]. In order to meet clinical and regulatory standards, medical-grade wearables adopted several strategies to deliver accurate temperature data, including monitoring at more reliable anatomical sites such as the axilla, which closely approximates core temperature when properly acquired [66], and developing algorithms to estimate core temperature from raw sensor signals. Accordingly, the average readings of estimated core temperatures and axillary temperatures were around 36.3 °C to 37.2 °C [40,62], which were mainly considered in studies that presented wearable data in clinical practice.

Unsurprisingly, skin temperatures directly provided by wearables at wearing sites, such as the wrist or chest, remain more variable in terms of validity and clinical utility. The mean skin temperatures were reported around 33.9 °C to 34.8 °C in reviewed studies [43,44], showing significant deviation from commonly used clinical values, particularly when determining fever. Therefore, several studies withheld skin temperature from clinical staff or discarded data from analyses [67-69], highlighting a cautious stance toward its clinical relevance. Some studies limited data collection periods, monitoring wrist skin temperature only during sleep to reduce daily activity influences [43]. However, studies have embraced more innovative strategies to actively use skin temperature, such as identifying elevated values as a predictive signal for fever [55], detecting abnormal circadian rhythms in patients with critical illness [49], and directly using its cutoff values to predict infection [44]. These methods view skin temperature as an independent output pathway of the thermoregulatory system, offering a potentially clinically useful route for its application. Notably, 1 study reported that both axillary and skin temperature emerged as reliable biomarkers, yet prioritized wearable-derived skin temperature over axillary measurements for its higher sampling frequency, continuous availability, and ease of future deployment [59]. Therefore, for clinical interpretation of wearable temperature data, health care providers should not be limited by absolute value comparability to traditional measurements but integrate trend-based and pattern-recognition approaches to expand clinical utility of wearable data across diverse monitoring contexts.

### Filtering Anomalies Before Using Temperature Data From Wearables

Although medical-grade wearables exhibit accuracy, prolonged usage in real-world clinical environments still presents significant data quality challenges arising from patient nonadherence, poor skin contact, device malfunction, sensor drift, and signal artifacts. Efforts have been made by manufacturers to assign quality scores to each temperature reading via wearables’ firmware [41,45,46,51]. However, the reliability of quality scores has not been adequately validated, and their calculations often lack transparency due to proprietary algorithms and patent protection. As indicated by Koenig et al [41], the wearables’ quality assessment was generally plausible, but it occasionally misclassified vital signs by assigning good quality to incorrectly measured data and poor quality to plausible extreme values. Therefore, studies in this review differed in their use of quality scores. We highly recommend that future studies validate quality scores before deciding whether to incorporate them.

Identifying anomalies should be guided by both data characteristics and clinical domain knowledge, as anomalies may still fall within the physiological boundaries yet represent clinically significant events [15]. The most commonly used method in the reviewed studies relied on thresholds to identify invalid temperature data, which has significant limitations. Removing axillary temperature readings below 35.5 °C may overlook inadvertent hypothermia in patients with critical illness [70] or patients who had undergone surgery [66]. Moreover, recent studies suggest that human basal body temperature has declined over the past century [71]. A systematic review calculated the normal range for axillary temperature to be 35.01 °C to 36.93 °C [72], indicating that a fixed 35.5 °C may not be able to account for individual variations in baseline temperatures. Additionally, the high variability in skin temperature makes it more challenging to determine reasonable thresholds for outlier removal. Instead, specialized artifact detection algorithms have been developed in a broader context. For example, Bardia et al [73] developed algorithms to identify adjacent temperature readings with slopes exceeding 0.08 or an absolute change greater than 0.25 °C, which effectively filtered intraoperative temperature artifacts. Future efforts should develop and incorporate such standardized data cleaning frameworks based on specific clinical contexts and professional knowledge, enabling personalized anomaly detection while preserving clinically relevant events.

After identifying anomalies in temperature data, handling incomplete datasets remains challenging. Most reviewed studies simply excluded cases with high missingness or deleted anomalous window segments. While retaining only valid data is feasible for basic statistical comparisons, removing data disrupts time-series signal continuity and limits specific feature extractions that require continuous data as inputs, such as entropy metrics for nonlinear dynamics [13]. On the other hand, the decision to impute missing data also requires careful evaluation. A study comparing imputation techniques for wireless continuous vital signs found that linear interpolation reduced feature bias (eg, 2-hour window mean and slope) relative to no imputation, whereas other techniques (eg, last observation carried forward and spline interpolation) increased bias, with imputation errors growing significantly with gap length [74]. Some advanced strategies have emerged as alternatives to imputation. For instance, dimensionality and size reduction based on intrinsic and target similarity can be used to eliminate features and samples that are highly sensitive to missing values [75], a principle that may extend to physiological time-series modeling. In addition, algorithms with built-in tolerance to missing data may improve model robustness and generalizability. Overall, we recommend selecting the appropriate preprocessing approach based on the specific downstream task, following careful assessment of potential bias.

### Advancing Temporal Pattern Mining and Interpretation for Wearables Data

Our review found that descriptive analytics remain the predominant approach for analyzing wearable-generated temperature data, with visual inspection of the trends and spikes in temperature curves being a common approach. This approach is intuitive, convenient, and easy to interpret in clinical practice, without requiring additional computational resources. However, it may not allow for detecting a specific pattern or predictive signals preceding infections [41]. In contrast, time series analysis and machine learning models for diagnostic or predictive applications were used in only one-third of the publications reviewed, indicating underutilization of these advanced analytical capabilities.

To leverage the hidden temporal patterns in high-frequency temperature data, machine learning and time series models represent an important methodological evolution. These methods extract temperature features that indicate circadian rhythm variations and pattern similarities, capturing subtle yet crucial indicators of infection that are not discernible through direct visual inspection. Beyond the temperature features identified in this review, studies have examined temperature dynamics in a broader context. For example, Papaioannou et al [76] assessed temperature complexity using wavelet transformation and multiscale entropy to classify patients with systemic inflammatory response syndrome, sepsis, and septic shock. Bhavani et al [77] identified 4 temperature trajectories from hourly recorded EHR data, revealing sepsis subphenotypes with distinct biomarker profiles. These advances highlight the need for further integration of advanced data analytics for temperature temporal pattern mining and knowledge discovery. These algorithms offer promising solutions to tackle data overload challenges by filtering meaningful and actionable information from massive wearable data; however, they still require further integration into computerized decision support systems to enable intelligent analysis and transform data into simpler and more usable forms.

### Integration of Wearable Devices Into Clinical Practice

We found that several wearables have been used for temperature monitoring as a supplement to standard nursing measurements, but their integration into routine clinical practice remains limited. The wearable systems were deployed independently, with only 1 study enabling alerts through EHRs [17]. This may stem from health systems’ requirement to establish a dedicated secure network for wearables, separated from the main network to prioritize data privacy [78]. However, this approach creates data silos. Although temperature is a key indicator for infection detection, final clinical diagnosis or treatment decisions require synthesis of wearable data with laboratory findings, clinical assessments, and patient history. Advancing system interoperability is essential to support comprehensive clinical decision-making rather than switching between systems to access fragmented data. Potential solutions include establishing standardized APIs through third-party integration platforms and developing plug-and-play interoperability standards [78]. Additionally, to streamline nursing workflow, more efforts are still needed to achieve automatic uploading of wearable data to nursing records, rather than requiring manual transcription of data from one screen to another. However, as previously discussed, such system integration must incorporate automatic artifact filtering algorithms to prevent recording erroneous data without professional oversight.

To better support clinical practice using wearables, we identified several gaps requiring further attention regarding data access interfaces, data latency, and data security. First, some studies deployed central monitoring systems at the nursing station, which enable simultaneous multipatient monitoring but prevent immediate access to real-time readings when assessing patients at bedside. A hybrid approach combining central and mobile interfaces would optimize both oversight and point-of-care accessibility. Second, some studies used batch uploads at fixed intervals, which conserve energy and extend battery life but may delay clinical reactions to patient deterioration. Transmission frequency must align with clinical context to ensure real-time data streaming, with transparent reporting enabling proper data latency assessment. Third, data security is a growing concern, yet only 10 studies reported specific protection strategies, with 50% (5/10) conducted in Europe under strict EU privacy regulations [20]. There remains a need for stronger adherence to safety and privacy regulations globally.

Overall, the clinical integration of wearables for fever and infection management remains predominantly at an early stage of maturity, aligning with existing evidence from digital health interventions for postoperative monitoring [79] and oncology care [20]. Although the scope of regulatory approval limited some devices’ clinical application, the reasons more frequently reported for not providing data to support clinical decision-making were insufficient data accuracy and limited real-time availability [53]. We also noted that certain devices (eg, SensiumVitals, Everion, and Gen2, listed in Multimedia Appendix 3) are not intended for use in critical care patients, potentially associated with reduced measurement reliability under unstable hemodynamic conditions. This explains their limited use in ICUs (Figure 2) and underscores the need for caution when using wearables for patients who are clinically deteriorating in general wards and avoiding sole reliance on wearable data for clinical decisions. Beyond technical limitations, there are systemic barriers to overcome before wearables can become widely accepted with full clinical integration, such as data ownership and privacy, data standards and interoperability, and reimbursement policies [80]. Additionally, the geographic concentration of reviewed studies in well-resourced areas highlights potential equity challenges, as disparities in device access, digital literacy, and infrastructure may limit scaled implementation and exacerbate health inequalities. Nevertheless, the emergence of wearables in clinical settings shows a developmental trajectory outlined in the maturity framework, which delineates the accumulation of evidence throughout the device lifecycle from initial prototype to full clinical integration.

### Future Directions

With the rapid iteration of wearable technology, novel devices are increasingly emerging. While existing evidence mainly focused on lightweight rigid wearables, we identified some textile-based flexible wearables for remote monitoring of infectious diseases in the literature search. However, these textile-based devices are still in the proof-of-concept [81], technical testing [82], and animal experimental stages [83], possibly reflecting the current technological bottlenecks in achieving clinical accuracy and limited maturity in regulatory approval and clinical validation [84]. This development reflects an evolution in wearable technologies toward softer, lighter, and safer sensing materials. In future studies, our maturity framework could serve as a tool to support health care providers in evaluating novel wearables’ readiness for clinical deployment and selecting suitable devices. Noncontact vital sign monitoring technologies, such as camera-based or robotic sensing systems, represent another emerging direction in continuous physiological surveillance in hospital environments [85] and may offer supplementary options for body temperature monitoring in special populations, such as neonates with fragile skin intolerant to adhesive sensors [86], although these technologies fall outside the scope of this review.

As wearables become increasingly used in clinical practice, nurses need to be better prepared for this transformation. Nurses are not only frontline users but also key researchers in this field, with nearly one-third of the studies reviewed being nurse-led. In future clinical deployment, large-scale data streams from wearables will require a robust infrastructure and storage capacity to prevent data loss or overwriting. These data could be incorporated into the clinical research data warehouse as a valuable source. Moreover, to harness the value of big data and AI, it is important for nurses to develop digital literacy and data science competencies to integrate nursing expertise into data interpretation. This will empower nurses to play an active role in wearable-driven health care transformation, rather than being passive end users of monitoring technology.

The observed lack of transparency highlights the need for reporting guidelines specific to clinical research using wearables. Our findings indicate low reporting rates on fundamental aspects, such as device grade (12/29, 41%) and regulatory status (10/29, 34%). While wearable data quality is crucial for appropriate clinical interpretation [87], many publications did not report data accuracy (11/29, 38%) and missing data (8/26, 31%). To improve rigor, the device grade, regulatory status, and clinical validation should be standard reporting elements. For feasibility and pilot studies, data transmission, latency, and loss rates could be taken into assessment and reporting. In predictive modeling research, reporting missing sensor data and any imputation methods is crucial. Data privacy and clinical accessibility could also be incorporated into reporting guidelines.

### Limitations of This Review

This review has several limitations. First, we focused specifically on the use of wearables for monitoring body temperature in fever or infection management. The assessment of maturity is limited to this specific area, and maturity stages may differ in other fields. Second, we did not assess the quality or risk of bias of the included studies, which means our findings are based on all available evidence, regardless of its methodological rigor. Third, we only included studies published in English or Chinese, which may have excluded evidence published in other languages. Additionally, only a random 20% of the records were screened and extracted independently by 2 reviewers. While this approach is methodologically acceptable [88], it remains a potential source of bias. Furthermore, we identified the roles of nurses from the main texts, author, and affiliation information. However, confirming the presence of nurses among the authors was not always possible due to the lack of explicitly stated nursing qualifications.

### Conclusions

This scoping review summarized ways of using temperature data from wearables across diverse clinical contexts, showing promise in optimizing fever or infection management in acute care hospitals, particularly when integrated with advanced data analytic approaches. However, the overall integration of wearables into clinical practice remains at an early stage of maturity. Key issues include data quality challenges, clinical interpretation of skin surface temperature, and interoperability between wearable systems and EHRs. Future efforts should prioritize standardized data cleaning frameworks and EHR interoperability, and facilitate clinical interpretation of wearable data to promote its active use in decision-making. These efforts will help to unlock the full potential of wearables in driving health care transformation.

## Data Availability

All data generated or analyzed during this study are included in this published article and its supplementary information files.

## Appendix

Multimedia Appendix 1 PRISMA-ScR checklist.

Multimedia Appendix 2 Search strategy.

Multimedia Appendix 3 Data extraction form.

## Abbreviations

CE: Conformité Européenne
CRS: cytokine release syndrome
EHR: electronic health record
EU: European Union
EWS: early warning score
FDA: US Food and Drug Administration
ICU: intensive care unit
JBI: Joanna Briggs Institute
NFC: near-field communication
OSF: Open Science Framework
PCC: population, concept, context
PRESS: Peer Review of Electronic Search Strategies
PRISMA-ScR: Preferred Reporting Items for Systematic Reviews and Meta-Analyses extension for Scoping Reviews
ROC: receiver operating characteristic
SpO2: peripheral oxygen saturation
WHO: World Health Organization
XGBoost: extreme gradient boosting
