# Supplementary material for: Continuous Temperature Monitoring via Wearable Devices for Fever or Infection Management in Acute Care Hospitals: Scoping Review of Clinical Implementation and Data Analytics
Source: JMIR Mhealth Uhealth. 2026 Sep 11;14:e89773. doi: 10.2196/89773 (PMC13615382; doi:10.2196/89773)
Supplement: Supplementary file 2 [file mhealth_v14i1e89773_app2.docx]

**Search Strategies**

**Databases: Medline, Embase, Web of Science, CINAHL, IEEE Xplore**

**1. Database: MEDLINE** (Ovid MEDLINE® ALL)

Original search date: February 24, 2025; Search update: July 06, 2026

| **Search framework** | **#** | **Search Strategy** | **Results** |
| --- | --- | --- | --- |
| **Concept**  *Wearable device* | **1** | **exp Wearable Electronic Devices/**  Fitbit.mp.  VitalPatch.mp.  TempTraq.mp.  iThermonitor.mp.  HEARThermo.mp.  Apple watch.mp.  Huawei watch.mp.  Oura Ring.mp.  SensiumVitals.mp.  ViSi Mobile.mp.  Aingeal.mp.  MiniMedic.mp.  Zephyr BioPatch.mp.  Equivital EQ02 Lifemonitor.mp.  Empatica.mp.  smartwatch*.mp. | 77,284 |
|  |  | Combine with ‘OR’ |  |
|  | **2** | wearable*.mp.  smart.mp.  mobile.mp.  portable.mp.  temperature.mp. |  |
|  |  | Combine with ‘OR’ |  |
|  | **3** | sensor.mp.  biosensor.mp.  thermometer.mp.  patch.mp.  watch.mp.  device.mp.  ring.mp.  wristband*.mp. |  |
|  |  | Combine with ‘OR’ |  |
|  | **4** | #1 OR (#2 AND #3) |  |
| **Concept**  *Body temperature* | **5** | **exp Body Temperature/**  body temperature.mp.  skin temperature.mp.  core temperature.mp. | 111,644 |
|  |  | Combine with ‘OR’ |  |
| **Concept**  *Fever* | **6** | **exp Fever/**  fever*.mp.  pyrexia.mp.  hyperpyrexia.mp.  febrile*.mp.  elevated body temperature.mp.  increased body temperature.mp.  hyperthermia.mp. | 298,644 |
|  |  | Combine with ‘OR’ |  |
| **Concept**  *Infections* | **7** | **exp Infections/**  **exp Inflammation/**  infectio*.mp.  inflammat*.mp.  bacteremia.mp.  SIRS.mp.  sepsis.mp.  septic.mp.  pneumonia.mp.  abscess*.mp.  fester*.mp.  suppurat*.mp.  purulent.mp. | 4,878,573 |
|  |  | Combine with ‘OR’ |  |
|  | **8** | #6 OR #7 | 4,980,276 |
| **Population**  *Patient* | **9** | **exp Patients/**  patient*.mp. | 7,963,605 |
|  |  | Combine with ‘OR’ |  |
| **Context**  *Acute hospital settings* | **10** | **exp Hospitals/**  hospital*.mp.  ward*.mp.  in-hospital.mp.  inpatient.mp.  acute care.mp.  emergency dep*.mp.  intensive care unit*.mp.  critical care.mp.  clinical.mp. | 6,277,881 |
|  |  | Combine with ‘OR’ |  |
|  | **11** | #4 AND #5 AND #8 AND #9 AND #10 | 306 |
|  | **12** | Filter: year="2013 -2025" | 159 |
| Search update | | Filter: year="2025 -Current" | 39 |

[mp=title, book title, abstract, original title, name of substance word, subject heading word, floating sub-heading word, keyword heading word, organism supplementary concept word, protocol supplementary concept word, rare disease supplementary concept word, unique identifier, synonyms, population supplementary concept word, anatomy supplementary concept word]

**2. Database: Embase** (via Ovid)

Original search date: February 24, 2025; Search update: July 06, 2026

| **Search framework** | **#** | **Search Strategy** | **Results** |
| --- | --- | --- | --- |
| **Concept**  *Wearable device* | **1** | **exp wearable device/**  Fitbit.mp.  VitalPatch.mp.  TempTraq.mp.  iThermonitor.mp.  HEARThermo.mp.  'Apple watch'.mp.  'Huawei watch'.mp.  'Oura Ring'.mp.  SensiumVitals.mp.  ViSi Mobile.mp.  Aingeal.mp.  MiniMedic.mp.  'Zephyr BioPatch'.mp.  'Equivital EQ02 Lifemonitor'.mp.  Empatica.mp.  smartwatch*.mp. | 170,214 |
|  |  | Combine with ‘OR’ |  |
|  | **2** | wearable*.mp.  smart.mp.  mobile.mp.  portable.mp.  temperature.mp. |  |
|  |  | Combine with ‘OR’ |  |
|  | **3** | sensor.mp.  biosensor.mp.  thermometer.mp.  patch.mp.  watch.mp.  device.mp.  ring.mp.  wristband*.mp. |  |
|  |  | Combine with ‘OR’ |  |
|  | **4** | #1 OR (#2 AND #3) |  |
| **Concept**  *Body temperature* | **5** | **exp body temperature/**  'body temperature'.mp.  'skin temperature'.mp.  'core temperature'.mp. | 120,324 |
|  |  | Combine with ‘OR’ |  |
| **Concept**  *Fever* | **6** | **exp fever/**  fever*.mp.  pyrexia.mp.  hyperpyrexia.mp.  febrile*.mp.  'elevated body temperature'.mp.  'increased body temperature'.mp.  hyperthermia.mp. | 666,749 |
|  |  | Combine with ‘OR’ |  |
| **Concept**  *Infections* | **7** | **exp infections/**  **exp inflammation/**  infectio*.mp.  inflammat*.mp.  bacteremia.mp.  SIRS.mp.  sepsis.mp.  septic.mp.  pneumonia.mp.  abscess*.mp.  fester*.mp.  suppurat*.mp.  purulent.mp. | 8,453,819 |
|  |  | Combine with ‘OR’ |  |
|  | **8** | #6 OR #7 | 8,598,039 |
| **Population**  *Patient* | **9** | **exp patient/**  patient*.mp. | 13,897,848 |
|  |  | Combine with ‘OR’ |  |
| **Context**  *Acute hospital settings* | **10** | **exp hospitals/**  hospital*.mp.  ward*.mp.  in-hospital.mp.  inpatient.mp.  'acute care'.mp.  'emergency dep*'.mp.  'intensive care unit*'.mp.  'critical care'.mp.  clinical.mp. | 16,199,768 |
|  |  | Combine with ‘OR’ |  |
|  | **11** | #4 AND #5 AND #8 AND #9 AND #10 | 1,732 |
|  | **12** | Filter: year="2013 -2025" | 1,424 |
| Search update | | Filter: year="2025 -Current" | 250 |

[mp=title, abstract, heading word, drug trade name, original title, device manufacturer, drug manufacturer, device trade name, keyword heading word, floating subheading word, candidate term word]

**3. Database: Web of Science Core Collection**

Original search date: February 24, 2025; Search update: July 06, 2026

| **Search framework** | **#** | **Search Strategy** | **Results** |
| --- | --- | --- | --- |
| **Concept**  *Wearable device* | **1** | TS=("Fitbit")  TS=("VitalPatch")  TS=("TempTraq")  TS=("iThermonitor")  TS=("HEARThermo")  TS=("Apple watch")  TS=("Huawei watch")  TS=("Oura Ring")  TS=("Oura Ring")  TS=("SensiumVitals")  TS=("ViSi Mobile")  TS=("Aingeal")  TS=("MiniMedic")  TS=("Zephyr BioPatch")  TS=("Equivital EQ02 Lifemonitor")  TS=("Empatica")  TS=("smartwatch*") | [520,407](https://www-webofscience-com.manchester.idm.oclc.org/wos/woscc/summary/22a99893-078c-4331-8328-e1ee22d6c2ee-014972f14e/relevance/1) |
|  |  | Combine with ‘OR’ |  |
|  | **2** | TS=("wearable*")  TS=("smart")  TS=("mobile")  TS=("portable")  TS=("temperature") |  |
|  |  | Combine with ‘OR’ |  |
|  | **3** | TS=("sensor")  TS=("biosensor")  TS=("thermometer")  TS=("patch")  TS=("watch")  TS=("device")  TS=("ring")  TS=("wristband*") |  |
|  |  | Combine with ‘OR’ |  |
|  | **4** | #1 OR (#2 AND #3) |  |
| **Concept**  *Body temperature* | **5** | TS=("body temperature")  TS=("skin temperature")  TS=("core temperature") | 68,268 |
|  |  | Combine with ‘OR’ |  |
| **Concept**  *Fever* | **6** | TS=("fever*")  TS=("pyrexia")  TS=("hyperpyrexia")  TS=("febrile*")  TS=("elevated body temperature")  TS=("increased body temperature")  TS=("hyperthermia") | 321,059 |
|  |  | Combine with ‘OR’ |  |
| **Concept**  *Infections* | **7** | TS=("infectio*")  TS=("inflammat*")  TS=("bacteremia")  TS=("SIRS")  TS=("sepsis")  TS=("septic")  TS=("pneumonia")  TS=("abscess*")  TS=("fester*")  TS=("suppurat*")  TS=("purulent") | 4,112,749 |
|  |  | Combine with ‘OR’ |  |
|  | **8** | #6 OR #7 | 4,295,166 |
| **Population**  *Patient* | **9** | TS=("patient*") | 9,153,225 |
| **Context**  *Acute hospital settings* | **10** | TS=("hospital*")  TS=("ward*")  TS=("in-hospital")  TS=("inpatient")  TS=("acute care")  TS=("emergency dep*")  TS=("intensive care unit*")  TS=("critical care")  TS=("clinical") | 6,409,979 |
|  |  | Combine with ‘OR’ |  |
|  | **11** | #4 AND #5 AND #8 AND #9 AND #10 | 299 |
|  | **12** | Filter: year="2013 -2025.2" | 205 |
| Search update | | Filter: year="2025.2 -Current" | 22 |

**4. Database: CINAHL Plus** (via EBSCO)

Original search date: February 24, 2025; Search update: July 06, 2026

| **Search framework** | **#** | **Search Strategy** | **Results** |
| --- | --- | --- | --- |
| **Concept**  *Wearable device* | **1** | **MH "Wearable Sensors+"**  Fitbit  VitalPatch  TempTraq  iThermonitor  HEARThermo  "Apple watch"  "Huawei watch"  "Oura Ring"  SensiumVitals  ViSi Mobile  Aingeal  MiniMedic  "Zephyr BioPatch"  "Equivital EQ02 Lifemonitor"  Empatica  smartwatch* | 24,487 |
|  |  | Combine with ‘OR’ |  |
|  | **2** | wearable*  smart  mobile  portable  temperature |  |
|  |  | Combine with ‘OR’ |  |
|  | **3** | sensor  biosensor  thermometer  patch  watch  device  ring  wristband* |  |
|  |  | Combine with ‘OR’ |  |
|  | **4** | #1 OR (#2 AND #3) |  |
| **Concept**  *Body temperature* | **5** | **MH "Body Temperature+"**  "body temperature"  "skin temperature"  "core temperature" | 16,916 |
|  |  | Combine with ‘OR’ |  |
| **Concept**  *Fever* | **6** | **MH "Fever+"**  fever*  pyrexia  hyperpyrexia  febrile*  "elevated body temperature"  "increased body temperature"  hyperthermia | 58,489 |
|  |  | Combine with ‘OR’ |  |
| **Concept**  *Infections* | **7** | **MH "Infection+"**  **MH "Inflammation+"**  infectio*  inflammat*  bacteremia  SIRS  sepsis  septic  pneumonia  abscess*  fester*  suppurat*  purulent | 744,946 |
|  |  | Combine with ‘OR’ |  |
|  | **8** | #6 OR #7 | 776,423 |
| **Population**  *Patient* | **9** | **MH "Patients+"**  patient* | 2,009,876 |
|  |  | Combine with ‘OR’ |  |
| **Context**  *Acute hospital settings* | **10** | **MH "Hospitals+"**  **MH "Hospital Units+"**  hospital*  ward*  in-hospital  inpatient  "acute care"  "emergency dep*"  "intensive care unit*"  "critical care"  clinical | 2,138,319 |
|  |  | Combine with ‘OR’ |  |
|  | **11** | #4 AND #5 AND #8 AND #9 AND #10 | 188 |
|  | **12** | Filter: Publication Year: 2013-2025 | 108 |
| Search update | | Filter: year="2025 -Current" | 13 |

**5. Database: IEEE Xplore**

Original search date: February 24, 2025; Search update: July 06, 2026

All Metadata

| **Search framework** | **#** | **Search Strategy** | **Results** |
| --- | --- | --- | --- |
| **Concept**  *Wearable device* | **1** | Fitbit  VitalPatch  TempTraq  iThermonitor  HEARThermo  "Apple watch"  "Huawei watch"  "Oura Ring"  SensiumVitals  ViSi Mobile  Aingeal  MiniMedic  "Zephyr BioPatch"  "Equivital EQ02 Lifemonitor"  Empatica  smartwatch* | 333,392 |
|  |  | Combine with ‘OR’ |  |
|  | **2** | wearable  smart  mobile  portable  temperature |  |
|  |  | Combine with ‘OR’ |  |
|  | **3** | sensor  biosensor  thermometer  patch  watch  device  ring  wristband |  |
|  |  | Combine with ‘OR’ |  |
|  | **4** | #1 OR (#2 AND #3) |  |
| **Concept**  *Body temperature* | **5** | "body temperature"  "skin temperature"  "core temperature" | 8,855 |
|  |  | Combine with ‘OR’ |  |
| **Concept**  *Fever* | **6** | fever*  pyrexia  hyperpyrexia  febrile*  hyperthermia | 4,536 |
|  |  | Combine with ‘OR’ |  |
| **Concept**  *Infections* | **7** | infectio*  inflammat*  bacteremia  SIRS  sepsis  septic  pneumonia | 19,677 |
|  |  | Combine with ‘OR’ |  |
| **Population**  *Patient* | **8** | patient* | 112,600 |
| **Context**  *Acute hospital settings* | **9** | hospital*  ward*  in-hospital  inpatient  "acute care"  "emergency dep*"  "intensive care unit*"  "critical care"  clinical | 156,350 |
|  |  | Combine with ‘OR’ |  |
|  | **10** | #4 AND #5 AND #6 AND #8 AND #9  Filter: Publication Year: 2013-2025 | 17 |
|  | **11** | #4 AND #5 AND #7 AND #8 AND #9  Filter: Publication Year: 2013-2025 | 58 |
| Search update | | Filter: year="2025-2026" | 15 |

In total,

Original search (February 24, 2025): 1,971 records were identified.

Search update (July 6, 2026): 338 additional records were identified.

6. **Grey literature search strategy**

Search date: 25/3/2025

| **Types of publications** | **Name of website** | **URL** | Serach terms used  (searched with different combinations) | **Additional records enrolled in screening** |
| --- | --- | --- | --- | --- |
| Dissertations | ProQuest Dissertations & Theses Global | [https://www.proquest.com/pqdtglobal?parentSessionId=iuF%2FeJ3PBqPHMg%2BTumFdFzRX3ZfdeH77RLkWBc5Y7uk%3D&accountid=12253](https://www.proquest.com/pqdtglobal?parentSessionId=iuF/eJ3PBqPHMg+TumFdFzRX3ZfdeH77RLkWBc5Y7uk=&accountid=12253) | wearable, temperature, fever, febrile, infectious, patient | 14 |
| Repositories | OpenGrey | [https://opengrey.eu/](https://opengrey.eu/) | wearable, temperature, fever, febrile, infectious, patient | 0 |
| Repositories | BASE – Bielefeld Academic Search Engine | <https://www.basesearch.net/> | wearable, temperature, fever, febrile, infectious, patient | 1 |
| Preprint | medRxiv | https://www.medrxiv.org/ | wearable, temperature, fever, febrile, infectious, patient | 3 |
| Preprint | [arXiv.org e-Print archive](https://arxiv.org/) | <https://arxiv.org/> | wearable, temperature, fever, febrile, infectious, patient | 0 |
| Preprint | bioRxiv | <https://www.biorxiv.org/> | wearable, temperature, fever, febrile, infectious, patient | 0 |
| Publications | World Health Organization | <https://iris.who.int/> | digital health, wearable device, body temperature, fever, infectious disease | 3 |
| Publications | Google | <https://www.google.co.uk/> | wearable, temperature, fever, febrile, infectious, patient; [site:.org/.edu/.gov] [filetype:pdf] | 3 |
| **Total** |  |  |  | **24** |
